# Supplementary material for: Are carbon emissions trading and green financial instruments synergistic? -Comprehensive quantitative research based on content analysis
Source: PLoS One. 2024 Mar 7;19(3):e0298601. doi: 10.1371/journal.pone.0298601 (PMC10919601; doi:10.1371/journal.pone.0298601)
Supplement: S1 Data — (ZIP) [file pone.0298601.s001.zip › Data/地级市Z_F工作报告.zip!/╣π╢1⁄2/║╙╘┤╩╨/└┤╫╘╣1⁄2╓┌║┼í╛┬φ┐╦╩2╛▌═°í┐_2017.docx]

各位代表：

　　现在，我代表市人民政府，向大会报告政府工作，请予审议，并请市政协各位委员提出意见。

　　一、过去五年工作回顾

　　五年来，我们在以习近平同志为核心的党中央坚强领导下，在省委、省政府和市委的正确领导下，在市人大及其常委会和市政协的监督支持下，攻坚克难搞建设，凝心聚力促发展，较好地完成了各项目标任务，全市经济社会发展迈上新台阶。

　　经济发展可圈可点。预计2016年，全市实现地区生产总值895.95亿元，比上年增长8.6%，五年年均增长（下同）10.3%；人均生产总值2.9万元，增长8.1%，年均增长9.4%；地方一般公共预算收入68.24亿元，增长1.1%，年均增长16.8%；完成固定资产投资650亿元，增长15.2%，年均增长22.3%；社会消费品零售总额538.53亿元，增长11.5%，年均增长12.6%；外贸进出口总额41.51亿美元，增长3%，年均增长9.1%；城镇居民人均可支配收入2.19万元，农村居民人均可支配收入1.18万元，年均分别增长9.3%和9.1%。

　　结构调整可圈可点。三次产业结构由2011年的13.6∶ 47.1∶39.3调整为11.9∶45.2∶42.9。2016年实现规模以上工业增加值360亿元，年均增长14.2%。高技术产业快速发展，五年新增高技术企业57家，2016年实现增加值87.2亿元，占全市规模以上工业增加值的24.2%。2016年，制造业投资占固定资产投资的34.5%，生产性服务业增加值占地区生产总值的14.7%，一般贸易进出口总额占外贸进出口总额的24.1%，民营经济增加值占地区生产总值的64.5%。

　　设施建设可圈可点。实施省、市重点项目491个，完成投资940亿元。增加等级公路1410公里，其中新建高速公路2条、151公里，通车里程达383公里，实现县县通高速；村道硬底化3000多公里；新建桥梁137座。新增35千伏及以上变电站9座、输电线路524.4公里。全市光缆线路达30.3万公里，建成3G/4G基站1.2万座，光纤覆盖达到94.5万户。

　　提质扩容可圈可点。中心城区建成区扩大到43.5平方公里，新建市政道路26条、现代城市综合体2个，市政设施和城市功能日趋完善。通过了国家卫生城市复审。入围中国最干净城市十强。江东新区成为第三批国家智慧城市试点。“西优”工程扎实推进，西环路及周边市政配套建设顺利。市区与东源县城连成一体，各县城框架进一步拉大，和平县城产城融合成效初显。创建了11个名镇、45个名村和4个省卫生镇、149个省卫生村。古竹镇入选首批中国特色小镇。

　　创新驱动可圈可点。获全国科技进步考核先进市称号。获省科学技术奖二等奖1项、三等奖5项。创建省级以上研发机构45家、市级研发机构81家。高技术企业达67家。专利授权量超过3300件。38个产品获广东省名牌产品称号。市高新区科技企业孵化基地建设顺利推进。

　　帮扶工作可圈可点。圆满完成两轮扶贫开发“双到”任务，帮扶修建村道789公里，完成农村低收入住房困难户住房改造12.44万户，“两不具备”村庄搬迁安置1.21万户，601个贫困村、6.65万户贫困户实现脱贫。精准扶贫工作开局良好，3.8万贫困人口实现脱贫。移民工作稳步推进，移民与非移民收入差距逐步缩小。深圳全面对口帮扶效果明显，2014年以来共投入帮扶资金53.5亿元，获全省第一轮对口帮扶工作（2014—2016年）评估考核第二名。

　　生态建设可圈可点。成为首批国家级生态保护与建设示范区，成功创建省林业生态市。建成国家级生态示范区1个、省级生态示范村镇园区31个、市级生态示范村152个。森林覆盖率达74.3%。

　　民生事业可圈可点。成功创建省教育强市。新、扩建中小学校356所，新增学位7万多个。新、扩建医院32间，乡镇卫生院标准化建设达标55间。市人民医院成功创建三甲医院。建成“三馆一园一剧院”等一批文化设施。转移农村劳动力23万人，安置下岗失业人员再就业4.8万人，推动就业困难人员再就业1.1万人。城乡居民基础养老金、居民医保财政补助标准、企业退休人员基本养老金水平五年分别提高100%、75%和43.5%。建设保障性住房、棚户区改造1.7万多套（户）。成功入选全国文明城市提名城市，连续3届获得全国双拥模范城称号。

　　政府效能可圈可点。网上办事大厅覆盖全市，省至村五级实现互联互通，市直部门网上全流程办理率91%、办结率71%，县区办理率70%、办结率83%。合并调整压减行政审批事项率、非行政许可审批事项率分别达47%和48%，投资项目审批时限大幅缩短。全面完成县区政府机构改革，全面实施政府权责清单制度。

　　五年来，我们重点抓好了七方面工作：

　　（一）始终把第一要务抓在手上，园区平台集聚效应凸显。推动工业园区扩能增效。五年投入开发资金130亿元，扩园42.81平方公里，园区开发面积达83平方公里，落户项目1041个，其中建成投产826个，新增规上工业企业300个。市高新区成为粤东西北首个国家级高新区。实现“五县三区”省级产业园（产业集聚区）认定“全覆盖”。电子信息、模具制造、新型材料等主导产业不断壮大，其中电子信息产业产值450亿元，五年增长2.8倍。加快万绿生态旅游产业园建设，建成巴伐利亚庄园等景区景点34个，新增国家4A级景区3家，引进了客天下等一批高端旅游文化项目。加快发展现代服务业。电子商务快速发展，互联网交易额年均增长30%。市高新区物流园、龙川铁路编组站物流园加快建设。我市成为省农村金融推广示范市和改革创新综合试验区，设立了前海股权交易（河源）中心、广州股权交易中心河源运营中心。加快发展现代农业，新增10个省级农业标准化示范区，灯塔盆地国家现代农业示范区成为国家农业创新与集成示范基地和国家农业科技园区。

　　（二）始终把设施建设抓在手上，区位环境条件明显改善。完成交通建设投资315.2亿元。汕湛高速（河源段，下同）、大广高速建成通车，汕昆高速、武深高速、河惠莞高速等加快推进，赣深高铁动工建设，河惠汕高速、广河高铁、杭广高铁、龙汕铁路等前期工作有序推进，改造国省道和县乡公路1000公里。河源进出境货运车辆检查场建成使用。大力发展民生水利，投入4.24亿元建设农村饮水安全工程148宗，解决了90余万农村居民饮水不安全问题；投入26.56亿元建设84宗中小河流治理项目，治理河长1240.51公里；投入10.1亿元完成水利示范县建设工程188宗。市区水源工程建设进展顺利。

　　（三）始终把城乡建设抓在手上，现代山水新城初具规模。积极推进“全域规划、多规合一”，编制了城市总体规划、中心镇总体规划等一系列规划。中心城区进一步扩容提质，改造市政道路26条，贯通“断头路”11条，完成背街小巷改造工程100多项；建成儿童公园、全民健身广场等一批公园、休闲广场，新增一批城市绿道；新、改、扩建农贸市场12个；建成迎客大桥。积极创建国家园林城市，扎实推进海绵城市试点建设。江东新区城市起步区全面动工建设。加强城市管理，打击“三违四抢”、整治“六乱”效果明显，城市“五化”水平提升。新农村建设加快推进。完成农村集体土地所有权确权登记发证工作，农村土地承包经营权确权颁证工作顺利开展。“特色小镇、美丽乡村”建设和宜居村镇“六个一”工程试点进展顺利。

　　（四）始终把改革创新抓在手上，新的发展动能正在形成。推进协同创新，建立河源广工大协同创新研究院、省科学院河源研究院，实施产学研项目161项。全市拥有省级工程技术中心43家、省级产业技术创新联盟1家、国家级孵化器培育单位1家。市高新区成为首批省级“双创”示范基地。安排技改资金8.7亿元，推动167家规上企业投资112.4亿元开展技术改造，产业水平不断提高。质量强市成效明显，11个产品获广东省名牌产品称号、474家企业通过质量管理体系认证；举办了首届“政府质量奖”。着力推进供给侧结构性改革，“三去一降一补”效果初显，为企业减负33.5亿元，清退4家“僵尸企业”，化解商品房库存165万平方米。深化行政审批制度改革，合并调整压减行政审批事项464项，社会投资项目审批时限从180天缩短至37天，政府投资项目审批时限从210天缩短至45天。推进“一门式、一网式”政务服务创新平台建设。基层公共服务综合平台全面建成。推进商事制度改革，市场主体净增57550户，三年增长74.8%。扎实推进国企改革，完成市直4家国企改制。“营改增”纵深推开，助推企业降本增效。在全省率先实施村（居）资金“双重”公示制度。

　　（五）始终把环境治理抓在手上，绿色优势更加巩固。深入实施新一轮绿化河源大行动，全市完成森林重点生态工程任务404.08万亩，建设生态景观林带391.3公里，建成森林公园86个、自然保护区1个、湿地公园8个，森林覆盖率提高2.5个百分点。新丰江水库入选国家重点支持保护湖泊，47个环保项目扎实推进。新建污水处理设施17座，完善配套管网88公里，全市污水日处理能力达32.6万吨。各县城建成生活垃圾填埋场，垃圾处理实现“一县一场、一镇一站、一村一点”。大力推进东江水环境综合整治，实施南粤水更清行动计划，东江河源段主要河流水质持续改善。实施“五大治污工程”，空气质量保持优良。完成省下达的节能减排任务。

　　（六）始终把民生福祉抓在手上，群众获得感明显增强。五年投入50多亿元，实现教育“五个全覆盖”，教育现代化水平和教育质量明显提高。进一步深化医疗卫生体制改革。新建市人民医院二期、市妇幼保健院二期、和平县人民医院，推进市中医院二期、紫金县人民医院建设。全市基本实现“一村一卫生站”，群众就医环境得到改善。传扬家训文化成为全国典型。市、县、镇、村文化设施基本达标。城镇新增就业每年超过4万人次，城镇登记失业率控制在2.42%以内。建成1426个村（社区）人力资源社会保障公共服务平台。城乡居民养老、医疗保险一体化，城乡居民基础养老金、居民医保财政补助标准、企业退休人员基本养老金水平分别提高到110元、420元和1701元。城镇、农村居民低保补贴水平分别提高到每人每月421元、197元以上，农村五保供养标准达到当地上年度农村人均纯收入的60%，残疾人生活津贴和重残护理补贴分别提高到每人每年1200元、1800元。因灾全倒户重建3146户。全面实施殡葬基本服务免费政策。深入开展“平安河源”建设，群众安全感连续多年排名全省前列。“六五”普法顺利实施，获评“全国法治宣传教育先进城市”。加强安全生产、应急管理、食药监管工作，未发生重大以上生产安全责任事故和较大以上食品药品安全事故，安全生产形势持续稳定好转。“双拥”工作深入开展，国防建设扎实推进。坚持每年办好“十件实事”，五年支出309.11亿元，办好了50件126项实事，着力解决群众的住房、就医、就业、教育、出行等问题。

　　（七）始终把廉洁勤政抓在手上，干事创业氛围更为浓厚。严格执行中央八项规定，全面加强党风廉政建设。深入开展党的群众路线教育实践活动、“三严三实”专题教育和“两学一做”学习教育，干部队伍作风建设得到加强。主动接受人大、政协及社会各界的监督，充分发挥监察、审计的作用，确保权力规范运行。认真开展“三赛”活动，实行“马上办”工作机制，深化行政体制改革，全面提升行政效能。

　　此外，民族、宗教、侨务、妇女、儿童、老龄、气象、打私、供销、史志、人民防空、防灾减灾等各项事业均取得了新成绩。

　　各位代表！五年的成绩来之不易！这是省委、省政府和市委正确领导的结果，是市人大及其常委会、市政协监督支持的结果，是全市人民团结拼搏、共同奋斗的结果。在此，我代表市人民政府，向全市广大干部群众，向各位人大代表、政协委员，向驻河源部队、消防官兵和各民主党派、人民团体、社会各界人士致以崇高的敬意和衷心的感谢！

　　在看到成绩的同时，我们也清醒地认识到当前面临的困难和问题。一是传统动力逐渐减弱，新的发展动能尚未形成，特别是工业化仍处在初级阶段，产业层次偏低、创新能力较弱，企业整体竞争力不强，处于微笑曲线的底端。二是用地条件、环境容量和劳动力矛盾日益突出，人才不足，资本市场不发达，交通网络有待完善，物流和生产成本较高。三是城镇化率较低，城镇要素集聚功能不强，辐射带动能力弱。四是民生欠账较多，基本公共服务供给能力不足，与人民群众的需求还有较大差距。五是部分干部担当意识不强，工作能力不足，干事创业作风与群众的期待有较大差距。对这些问题，我们将高度重视，认真加以解决。

　　二、今后五年的发展目标和任务

　　刚刚结束的市第七次党代会，为河源未来5年描绘了美好蓝图。我们一定要瞄准目标，把握大势，因势而谋抓机遇，顺势而为聚动能，乘势而进促发展。只要我们把思想和行动聚焦聚力到中央、省的战略决策和市委的工作部署上来，不偏离、不懈怠、不折腾，就一定能把方向定得更准，把任务抓得更牢，把举措落得更实，确保全面小康目标的实现。

　　今后五年政府工作指导思想是：高举中国特色社会主义伟大旗帜，紧密团结在以习近平同志为核心的党中央周围，坚决贯彻党的十八大和十八届五中、六中全会精神以及习近平总书记系列重要讲话精神，落实省委、省政府和市委的决策部署，牢固树立和贯彻落实新发展理念，适应把握引领经济发展新常态，以稳中求进、好中求快为工作总基调，以深入推进供给侧结构性改革为主线，按照大力实施“两大战略”和全力打好“两大战役”的要求，抢抓“两大机遇”，坚持产业支撑、坚持交通优先、坚持改革创新、坚持生态建设、坚持改善民生、坚持依法治理，努力实现在粤东西北率先振兴发展。

　　今后五年经济社会发展主要目标是：到2018年，实现地区生产总值1100亿元，地方一般公共预算收入80亿元，居民人均可支配收入2万元，与全省同步全面建成小康社会。到2021年，全市经济社会实现振兴发展，地区生产总值突破1500亿元，地方一般公共预算收入突破120亿元，居民人均可支配收入2.6万元。产业发展水平大幅提升，城乡基础设施日臻完善，生态宜居环境不断优化，社会保持和谐稳定，民主法制更加健全，人民幸福指数持续提高。初步建成珠江东岸绿色新兴产业集聚地、粤北赣南区域性综合交通枢纽、岭南健康休闲旅游名城和现代生态山水园林城市。

　　实现上述目标，我们要紧密结合市第七次党代会的战略部署，始终坚持“一二三四五”工作思路：

　　突出一条主线。突出以供给侧结构性改革为主线，瞄准短板做加法，瞄准过剩做减法，瞄准创新做乘法，瞄准放活做除法，推动“三去一降一补”取得实质性进展，深入推进产业结构调整和转型升级，大力发展实体经济。

　　实施“两大战略”、打好“两大战役”。深入实施创新驱动发展战略和全面融入深莞惠（3+2）经济圈，加快形成以创新为主要引领和支撑的经济体系和发展模式，加快推进新旧动能转换，着力构建创新型经济格局。突出打好产业发展大会战和精准脱贫攻坚战，以加快与深圳产业共建为切入点，以实施“四个倍增”计划为着力点，以精准扶贫为落脚点，加快河源发展，全面建成小康。

　　紧扣“三大抓手”。始终紧紧扭住交通基础设施建设、产业园区扩能增效、中心城区提质扩容不放松，充分发挥交通基础设施的先导作用、产业的支撑作用、城市的承载作用，为加快振兴发展奠定坚实基础。

　　着力做到“四个提升”。以项目建设为抓手、以改革创新为动力、以生态优势为依托、以改善民生为根本，着力提升项目建设、改革创新、生态保护和改善民生的工作水平，全力推进河源各项事业协调发展。

　　加快推进“五个一体化”。加强深河合作，主动对接深圳“东进”战略，实施河源“南融”行动，加快推进与深莞惠在新兴产业、交通运输、生态农业、环境保护和社会事业的“五个一体化”建设，有效借智借力，全面提升河源综合竞争力。

　　三、2017年重点工作安排

2017年是新一届政府的开局之年，是全面建成小康社会决胜阶段的关键一年。今年经济社会发展的主要预期目标是：全市地区生产总值增长8.7%，规模以上工业增加值增长10%，固定资产投资增长15%，社会消费品零售总额增长10%，对外贸易进出口总额增长3%，地方一般公共预算收入增长5.5%，居民人均可支配收入增长10%，居民消费价格总指数控制在3%以内，人口自然增长率控制在11‰以内，完成省下达的节能减排任务。

　　为实现上述目标，重点做好九方面工作：

（一）聚焦产业发展，着力增强经济实力。

以产业发展为引擎，努力构建产城融合、产业集聚的特色产业体系，加快推动协同发展、集聚发展。

　　狠抓产业园区建设。加大扩园力度，投入30亿元，扩园10平方公里以上，其中共建园区投入15亿元，扩园5平方公里。用好深圳对口帮扶资金和政策性银行贷款，重点推进深河产业城A区产业园建设。充分发挥水资源优势，继续推进水产业园建设。围绕先进制造业、现代服务业，加大招商引资、引技、引智、引才力度。开展定向招商、委托招商，加强与深圳等项目溢出地的信息互通，建立招商引资项目库，力促一批项目落地投产。全市园区实现新引进项目100个以上、新建设项目50个以上、新投产项目50个以上，园区工业增加值、税收分别增长10.5%、10%。

　　加快产业共建集聚。积极落实深圳河源产业共建合作协议，制订全方位扶持政策，吸引深圳优势产业、优质企业把生产和成果转化环节落户河源，打造“深圳总部+河源基地”“深圳研发+河源孵化”产业合作共建模式。争取引进不少于70个产业共建项目，合同投资额300亿元以上。注重主导产业集聚，引进培育电子信息、机械模具等新兴产业项目，推动各园区确立1-2个主导产业集群，打造环珠三角新兴产业集聚地。以中兴通讯项目为带动，加快引进上下游产业，打造千亿级电子信息产业集群。实施工业转型升级三年攻坚战行动计划，加快传统优势产业转型升级，全年规上技改企业达到70家，完成技改投资58亿元。

　　着力发展现代服务业。实施现代服务业发展规划，完善现代服务业扶持政策。加快万绿生态旅游产业园建设，推进创建“省级全域旅游示范市”和万绿湖创5A级景区工作，推动智慧旅游城市建设。力促客天下客家文化小镇、康泉十八二期建成开业，巴伐利亚庄园二期建设加快进度。推进霍山景区二期、林寨古村二期等旅游项目建设。培育发展健康养老、文化教育、体育健身等新兴消费热点。加快龙川铁路编组站物流园、市高新区综合物流园等物流项目建设。深入推进质量强市工作，积极打造区域品牌。培育壮大电子商务、文化创意、互联网金融等新型业态。

　　推动区域协调发展。坚持产业发展不动摇，推动县域经济发展提速，促进区域协调发展。突出园区主战场，以产兴城、以城促产、产城融合，促进产业园区与城镇建设有机衔接、协同发展，把产业园区建设成为产业新城、城市新区。引导企业向园区集聚发展，各县区产业园至少引进1-2个投资10亿元以上的优质项目，着力培育1-2个特色主导产业。鼓励县区大力发展食品饮料、绿色产品制造业，加快资源优势转化为经济优势。

　　（二）聚焦实体经济，着力做好供给侧加减法。

以振兴实体经济为抓手，把供给侧结构性改革放在更加突出的位置，在要素保障上做“加法”、在成本调控上做“减法”。

　　深化供给侧结构性改革。关停6家“僵尸企业”。完成省下达的淘汰落后产能任务，重点行业产能利用率达到合理水平。逐步化解现有房地产库存，全市商品房库存规模控制在449万平方米以内。推动金融去杠杆，确保金融机构杠杆率控制在合理区间。提高政府投资的有效性和精准性，有效增加公共产品和公共服务供给，补齐发展短板。

　　全力推动降本增效。打好“降成本”组合拳，帮助企业降低成本，包括降低制度性交易成本、税费负担、社会保险费、财务成本、电力价格、物流成本等，取消不必要的证明和手续，加快基础性关键性改革，减少制度和政策羁绊，为企业打造低成本、高效率的发展环境。

　　强化资源要素保障。大力清理批而未供、供而未用及低效用地，最大限度为重大产业项目预留用地空间。坚持财政引导和向上争取并重，切实用好中央、省各类鼓励扶持政策，撬动金融机构和社会资金加大对产业项目的投入。积极创新投融资体制机制，通过拨改投、债转股等方式扩大有效投资、降低金融风险。规范发展各级政府融资平台，增强融资能力。

　　积极发展民营经济。完善和落实扶持民营经济发展的政策措施，鼓励社会力量规划建设各类孵化功能较强的创业、创新基地。加快中小企业公共服务平台建设，提升综合服务能力。拓宽民间资本投资渠道，引导民营企业参与市政基础设施和政府投资项目建设。完善中小微企业融资机制，推动各县区建立信贷风险补偿机制，加大对中小微民营企业信贷支持。利用资本市场，拓宽融资渠道，支持民营企业做大做强。

　　（三）聚焦交通建设，着力提升区位优势。

　　突出以交通为重点，加快建设面向未来的基础设施，努力为振兴发展提供有力支撑。

　　推进交通基础建设。谋划建设更多连接珠三角、大内陆和出海港的快速通道。推进“五纵四横”高速公路网建设，力争汕昆高速龙连段、武深高速连平段年内建成通车，河惠莞高速加快建设，河惠汕高速纳入省“十三五”规划。全力配合赣深高铁建设，开展广河高铁、龙龙高铁、龙汕铁路前期工作。加强新编国、省道干线公路升级改造，推进国道G205热水至埔前段改线（东移）、河紫路升级改造等工程建设。加快建设通用机场、水上机场。

　　推进其他设施建设。加快河源电厂二期建设。完成中心村农村电网改造升级，促进城农网协调发展。加快高速宽带网络和云计算、大数据、物联网等信息基础设施建设，推广“三网融合”，实现城乡4G信号全覆盖、公共场所无线局域网全覆盖，打造“宽带河源”“光网河源”“数字河源”。推进中小河流治理、病险水库除险加固，完善江河水库防洪体系，提高防灾减灾能力。抓好“水利示范县”建设，继续实施“村村通自来水”工程。推进全国水生态文明城市建设。

　　（四）聚焦提质扩容，着力推进新型城镇化。

以提质扩容为核心，以新型城镇化为带动，把城乡建得更美，提升群众的归宿感和幸福感。

　　突出规划引领建设。完善城乡规划编制，推进规划“两个全覆盖”。制定“十三五”近期建设规划，为城镇化建设提供指引。实现控制性详细规划编制县城和中心镇覆盖率100%、其他乡镇60%，村庄规划编制覆盖率80%的目标。健全管理制度，制定城乡规划建设考核指标体系，严格实行规划实施责任追究制度。

　　做大做优中心城区。加快江东新区起步区建设，规划建设“高铁新城”。深入实施“西优”工程，加快推进双下湿地公园建设及双下路片区棚户区改造，打造美丽宜居西片区。新、改、扩建24条道路，重点改造提升中山大道、文明路、长安街和东环路等市政道路，贯通红星东路、纬六东路等“断头路”和小江桥下穿通道，启动纬十四路跨东江大桥、庄田新丰江大桥等桥梁建设。推进“老、旧、散”小区综合整治。加强和规范小区物业服务管理。实施宜居社区创建工作。加快城南、城北水厂建设，完成市区水源工程。推进海绵城市建设。综合运用金融、土地、财税、投资等手段，引导房地产市场健康发展。

　　提升城镇化水平。优化总体空间布局，着力构建“县城—中心镇—镇”城镇化体系。坚持城镇、交通、产业、生态和动力“五链融合”，逐步发展功能完善、特色鲜明的城乡新区。突出“特、强、小、美”，扎实开展“特色小镇、美丽乡村”建设。大力培育各具特色的工业强镇、旅游名镇、商贸重镇、农业大镇，推动农村转移人口就近、就地城镇化。

　　创建国家园林城市。对照国家园林城市标准，尽快补齐短板，顺利实现创建目标。重点改造提升一批现有公园，启动东江湾公园二期等公园建设，丰富内涵，完善设施；推进市区小游园、市植物园、石峡老垃圾填埋场生态修复工程、粤赣高速公路市区和城北出入口景观工程等建设；开展林荫道路和绿道网建设；建立园林绿化信息化管理系统，全面提升城市园林绿化和管护水平。

　　深化城市综合治理。加快市场秩序和城市管理综合执法体制改革，整合市政公用、市容卫生、园林绿化、城市管理执法等职能，增强依法治城能力。理顺市区管理体制，强化属地化管理。大力发展公共交通，加快停车场建设，努力解决交通拥堵、停车难等问题。大力整治城市“六乱”，提高“五化”水平。

　　（五）聚焦改革创新，着力增创发展优势。

以改革创新为动力，把跨越发展的基点放在创新上，在改革创新、区域合作中实现新旧动能转换。

　　推进重点领域改革。深化国企改革，引导非公有制企业参与国企改革，鼓励发展混合所有制经济。深化行政审批制度改革，重点抓好投资项目审批改革，提高审批效率。深化商事制度改革，探索多许可合并，完善市场监管机制。深化财税体制改革，实施中期财政规划管理，实现零基预算改革全覆盖，完成基本公共服务均等化综合改革试点工作。强化市场监管体系和社会信用体系建设，建立失信惩戒、守信激励机制。继续推进收入分配制度、户籍制度、司法、教育、就业和社会保障、安全生产等领域改革。

　　促进创新驱动发展。推进河源广工大协同创新研究院和省科学院河源研究院建设。大力引进高技术企业和科研机构，新增一批省级创新型企业、企业技术中心和工程技术研究中心。推动科技金融融合发展，加快广东科技金融综合服务中心河源分中心建设。推动“互联网+”应用，力争建成互联网创新孵化基地1个、产值超5亿元的智能制造产业集聚区1个，培育一批创新型互联网中小企业。强化知识产权保护，专利申请量增长15%以上。

　　强化人才支撑作用。推动人才优先发展措施有效落地，细化配套政策，着力培育和引进创新创业科研团队、急需紧缺人才、杰出人才和领军人才。推进职业教育现代化，创新和完善产教融合、校企合作、工学结合的人才培养模式，依托本地大中专院校，加强与省内外高校和专业培训机构合作，培养服务河源发展的适用性人才。重视企业家队伍建设。健全人才公共服务体系，优化人力资源配置，营造良好的人才发展环境。

　　（六）聚焦区域合作，着力推进“南融”行动。

　　主动对接珠三角、承接珠三角辐射，积极在推动粤东西北与珠三角一体化进程中发挥交通先导、产业对接、园区互补作用。

　　大力推进深河融合。引导深圳金融资本和社会资本参与河源基础设施项目和江东新区起步区建设，推进前海股权交易（河源）中心建设。落实两市农产品全链条式质量安全监管合作联动机制，共同认证挂牌一批深圳“菜篮子”河源生产基地。争取深圳加大对我市教育医疗卫生的帮扶力度，推动两市异地养老服务合作，推进两市医疗、养老保险、住房公积金缴存异地互认。

　　大力推进“3+2”合作。加快融入深莞惠（3+2）经济圈，主动参与大珠三角经济区和珠三角湾区城市群建设。积极推进五市物流运输信息共享平台建设、东江航道河源至惠州段升级改造，着力承接珠三角物流供应链分拨功能，进一步加快交通建设互联互通。推动现代生态农业融合发展，引导和支持珠三角企业在我市设立农副产品采购中心、建设农业生产加工基地。共享五市优质旅游资源，打造区域性旅游精品线路和产品。加快民生领域制度有效衔接，共建共享优质生活圈。

（七）聚焦绿色发展，着力提升生态优势。

以绿色发展为引领，做好治山理水的生态文章，让湖泊常清、江河长流、山城相依、田园相守的美好图景世代相传。

　　加强林业生态建设。强化主体功能区分区管控和生态控制线、林业生态红线划定管理。继续实施“绿化河源大行动”，推进重点林业生态工程建设，完成碳汇造林12.88万亩；推进生态景观林带工程建设，完成2605亩建设任务；推进森林进城围城工程建设，新增一批森林公园。强化生态功能区和自然保护区建设管理，提升森林生态功能。抓好速生桉树林改造和森林防火工作，加强林地资源保护利用。

　　严格实施环保制度。落实最严格水资源管理举措，全面实行“河长制”管理。继续推进东江水环境综合整治、实施南粤水更清行动计划，消除城市建成区黑臭水体。继续大力实施“五大治污工程”，抓好大气污染源治理。扎实推进重金属污染防治和土壤污染综合防治。严厉打击违法采石、采矿等行为，开展废弃矿区的污染治理和生态环境综合修复。积极实施农村环境保护行动计划，加强农村面源污染防治。完成“黄标车”淘汰任务。

　　扎实推进节能减排。推广新能源汽车、绿色建筑和建材的应用，支持既有建筑节能改造。鼓励和引导企业发展循环经济，推进省低碳试点市工作，开展碳排放权交易试点，探索建立碳汇交易平台。建立环境污染责任保险机制。严格执行建设项目主要污染物排放总量指标前置审核制度，控制污染物新增量。大力实施减排措施，加强减排工程运行监管，推进污水处理设施建设。

　　强化区域环保合作。探索建立区域水环境污染联防联治机制，构建东江水环境“一江同治”格局。强化大气污染联防联控，推动重点区域监测信息共享平台建设，建立区域大气污染事故预报、预警和应急处置机制。实行环保区域间联合、交叉执法。推进各地危险废物转移处置合作。建立企业环保信息区域共享和联合惩戒机制，完善企业环境信用评价和违法企业“黑名单”制度。

（八）聚焦精准扶贫，着力推进“三农”工作。

以精准扶贫为契机，努力促进农民增收，促进农业农村提升发展质量，实现“三农”工作的新发展。

确保扶贫取得实效。继续实施精准扶贫精准脱贫三年攻坚战行动计划，大力推进产业扶贫、光伏扶贫、电商扶贫，特别是引导支持贫困户因地制宜发展特色种养业。建立小额贷款基金，为贫困户发展产业提供贷款。加强技能培训，促进贫困劳动力转移就业。盘活贫困村的生产要素，使资源变资产、资金变股金、农民变股东，促进贫困人口增收。抓好兜底保障政策落地见效，实现最低生活保障制度和扶贫开发政策有效衔接，落实好贫困人口教育、医疗、住房“三保障”政策。建设覆盖农村低收入群体的大数据库，提高精准扶贫信息化管理水平。鼓励保险机构在缓解贫困群众因病致贫、因病返贫问题上发挥作用。

　　提高农业发展质量。深入推进农业供给侧结构性改革。推动农业“接二连三”，形成融合发展的高效价值链。健全农业技术推广、创新体系，实施农业品牌战略，提高农业质量、效益和竞争力。健全农副产品流通体系，加快市农副产品批发中心建设。以打造特色小镇为平台，以优质农林食品加工产业为重点，针对珠三角消费市场，培育一批农产品精深加工企业。加快灯塔盆地国家级现代农业示范区核心区建设。推动国家十大精准扶贫工程构树种养一体化项目落地。落实耕地保护责任，加强高标准基本农田建设，稳定粮食生产。做好第三次全国农业普查工作。

　　促进农民持续增收。培育新型职业农民。培育壮大新型经营主体，引导采取保底收购、股份分红、利润返还等方式，建立紧密型利益联结机制，增加农民经营性收入。扩大政策性涉农保险覆盖面，加快农村普惠金融示范村建设。促进农民获得更多就业机会，增加工资性收入。

　　实施农村提质工程。继续实施农村人居环境综合整治、建设“美丽乡村”三年行动计划，今年村庄污染整治覆盖率达到60%以上。深入开展宜居村镇“六个一”创建活动。继续推进水库移民美丽家园示范点建设。加快农村土地承包经营权确权登记颁证，深化农村集体产权制度改革，激发农村经济社会发展活力。

（九）聚焦公共服务，着力增进人民福祉。

以公共服务为重点，想群众所想，急群众所急，解群众所困，全面提升群众获得感。

　　推进教育现代化。积极推进教育现代化先进市创建工作，努力办人民满意的教育。加快推进教育优质化、均衡化发展，着力提高学前教育普惠性、公益性，增加市区和县城义务教育学位供给，深入实施振兴初中行动计划，提升农村地区教育水平，推动普通高中特色发展，促进职业教育内涵提升。大力实施教育信息化工程和“强师工程”。加快筹建本科院校。

　　大力创建卫生强市。完善基层医疗卫生机构运行机制，推进县镇村一体化管理。加强县级医院和基层医疗卫生机构服务能力建设，推动各县区建设二甲医院，提高县域内住院率。大力推进市人民医院等市属医院能力建设。建立健全分级诊疗体系。健全城乡医疗保险机制，完善药品供应保障体系。强化重大传染病防控，全面提升突发公共卫生事件应急处置能力。健全新生儿出生缺陷综合防控体系，提升计划生育服务管理水平。

积极发展文体事业。全力争创全国文明城市。深入开展“传扬家训·崇尚十德·日行一善”等主题活动，提升群众思想道德素质和科学文化素质。组织举办各类文化活动100场。加大乡镇文明建设力度，实现30%以上达到文明镇街的标准。提升20间乡镇（街道）综合文化站等级。大力推进省级公共文化服务体系示范区创建工作。鼓励文艺创作，扶持精品力作出版展演。支持文体创意产业发展，引资建设体育生态产业园。开展自然村落历史人文情况普查，加强文化遗产保护利用。推进市科技馆新馆、新丰江水库移民纪念馆和市、县区数字档案馆（室）建设。

　　深入开展社会治理。推动“互联网+社会治理”，加强立体化社会治安防控体系建设，继续深化打击突出刑事犯罪“飓风”专项行动，严厉打击黄赌毒、“两抢一盗”、电信诈骗和农村黑恶等违法犯罪行为。启动病残吸毒人员收治场所建设。加强电子商务、寄递物流等新业态和流动人口、重点人群动态管理和服务。治理影响安全生产、损害生态环境、危害食品药品安全、破坏网络安全等问题。增强防灾应急处置、消防应急综合救援和供电、通信设施抗灾能力。推进平安创建工作，深入治理社会矛盾，建立社会敏感问题预防处置体系，健全群众合理表达诉求机制。加强恐龙地质遗迹保护法律法规和科学知识的宣传教育，提高公民的恐龙地质遗迹保护意识。积极创建全国“七五”普法先进城市。

　　提升社会保障水平。扶持就业创业，落实失业保险援企稳岗政策。实施“互联网+创业”，建设创客空间等新型创业服务平台，推动大众创业、万众创新，城镇登记失业率控制在3%以内。实施全民参保登记计划，提高社会保险覆盖率。稳步推进机关事业单位养老保险制度改革。健全工资正常增长机制，完善最低工资标准调整和工资支付保障制度。加强社会救助体系建设，全面建立临时救助制度。启动创建第十一届全国双拥模范城工作。

　　今年，我们将继续突出办好十件民生实事：

　　一是提高底线民生保障水平。提高城镇和农村低保人均补差标准、农村特困人员救助供养标准、城乡医疗救助补助标准、孤儿基本生活最低养育标准、残疾人生活津贴和重残护理补贴标准等，确保不低于省定标准。下调困难群体大病保险起付标准，提高报销比例，不设年度最高支付限额。特困供养人员起付标准下调不低于80%，报销比例达到80%以上；建档立卡的贫困人员、最低生活保障对象起付标准下调不低于70%，报销比例达到70%以上。二是加大扶贫济困力度。实施相对贫困村“一村一品”产业推进行动，支持贫困人口参与度高的10个农业示范基地建设。全面落实建档立卡贫困人口“两兜底三保障”政策，对符合五保低保条件的贫困人口全部落实兜底扶持政策。对仍住危房的贫困户全部纳入住房改造计划。全额资助贫困人口参加城乡居民基本医疗保险，并纳入重特大疾病救助范围，对各项医保政策报销后的合规医疗费用按照不低于70%给予报销。落实建档立卡贫困学生生活补助政策，确保按省定标准落实到位。三是改善基层医疗卫生设施。完成20间乡镇卫生院标准化建设。启动东源县人民医院新院建设，启动东源县、紫金县各1间中心卫生院升级改造为县第二人民医院。四是提升教育保障水平。推进深河中学、公园东学校建设。启动河职院实训楼四期和学生活动中心建设，启动新、扩建源南镇学校、源城区特殊教育学校、东埔街道中心幼儿园、江源小学、城南小学、江东实验学校、联新小学等一批学校。加强学生资助管理工作，义务教育寄宿制公办学校公用经费补助从农村扩大到城市，补助标准提高到每生每年250元；对城乡义务教育家庭经济困难寄宿生，按小学每生每年1000元、初中每生每年1250元标准给予生活费补助；资助学前教育困难家庭幼儿1.2万人。将山区和农村边远地区学校教师生活补助标准提高到每人每月不低于900元。五是大力扶持创业就业。支持建设1个市级示范性创业孵化基地。加强劳动力技能培训，补贴参加技能晋升培训的劳动者1万人次；实施创业培训1800人，促进创业1800人，城镇新增就业3.5万人，帮扶失业人员再就业7000人、就业困难人员再就业1000人。六是改善农村生产生活条件。支持建设新农村公路300公里。完成农村危房改造1.1万户。将源城区、江东新区纳入普惠金融村村通试点范围，实现试点区全面建成综合征信中心，信用村全覆盖。七是加强公园广场建设。推进客家文化公园后期建设，升级改造市区文化广场、茶山公园和笔架山公园，启动东江湾公园二期项目和市民广场建设；推进和平县福和公园建设，加快龙川县“一江两岸”景观带、江滩公园、江边公园、苏区公园等项目规划建设。八是强化住房保障。全市新开工棚户区改造1751套（户）；基本建成各类保障性住房、棚户区改造511套（户），其中公共租赁住房172套、棚户区改造339户。九是加大环境治理力度。加速推进五县10座生活垃圾无害化处理场项目建设运行。完成在建10个、新建20个乡镇污水处理厂建设。整治东埔河黑臭水体和4条水质较差水体（三王坝、金竹沥、黄沙河、高埔小河）。村庄保洁覆盖面达90%以上，农村生活垃圾有效处理率达85%以上。新增市级生态示范村50个以上。完成农村人居环境综合整治年度任务。十是加强公共安全保障。推进智慧食药监项目建设；食品抽检和药品评价性抽验合格率分别达到90%和98%以上。实施平安气象工程，全市新建气象站点16个，升级改造22个，完善突发事件预警信息发布系统，建设超级生态气象站。全年消减地质灾害点50处以上。全面完成综治中心+网格化+信息化建设任务。

　　此外，将进一步做好民族、宗教、统计、打私、老龄、关工、对外、侨务、史志、基层政权等工作。

　　四、全面加强政府自身建设

　　切实增强政治意识、大局意识、核心意识和看齐意识，结合“两学一做”学习教育，建设法治、务实、高效、廉洁政府。

　　持续改进工作作风。严明政治纪律和政治规矩，营造风清气正的政治生态。全面落实党内监督的制度和责任，落实党内各项制度，保证领导干部忠诚干净担当。深入开展民主评议政风行风活动，坚决整治不作为乱作为。强化队伍建设，打造一支有担当、有定力、有责任、有作为的干部队伍。

　　深入推进依法行政。坚决执行市人大及其常委会的决议和决定，自觉接受市人大、市政协的监督，广泛听取各民主党派、工商联、无党派人士和各人民团体的意见。加强审计等监督工作。落实重大行政决策合法性审查制度，推进行政决策科学化、民主化、法治化。全面落实行政执法责任制。依法实施政务公开。

　　着力提升政府效能。深化简政放权、放管结合、优化服务改革，继续精简行政审批事项，完善负面清单、权力清单和责任清单，优化政府职能配置。推进“互联网+政务服务”，加快“一门式、一网式”政务服务体系建设，提高行政效能，着力打造全天候政府，努力营造“四最四低”的兴业环境。

　　切实加强廉政建设。坚持不懈推进政府系统党风廉政建设和反腐败工作。落实中央八项规定，坚决防止“四风”反弹，严控“三公”经费。完善土地出让、工程建设、产权交易、政府采购等制度，加强公共资金、公共资源、国有资产监管，严肃查处违纪违法案件。加强党风廉政教育，推进廉政文化建设，筑牢拒腐防变的思想道德防线，努力做到干部清正、政府清廉、政治清明。

各位代表！蓝图已绘就，扬帆正当时。让我们紧密团结在以习近平同志为核心的党中央周围，在省委、省政府和市委的坚强领导下，践行新理念、把握新机遇、激发新活力，全力开创河源振兴发展、全面奔康的新局面！
